# False-Positive Results of SARS-CoV-2 RT-PCR in Oropharyngeal Swabs From Vaccinators

**DOI:** 10.3389/fmed.2022.847407

**Published:** 2022-06-10

**Authors:** Xiang-Qi Kong, Yong-Jing Wang, Zan-Xi Fang, Tian-Ci Yang, Man-Li Tong

**Affiliations:** ^1^Center of Clinical Laboratory, School of Medicine, Zhongshan Hospital, Xiamen University, Xiamen, China; ^2^Xiamen Clinical Laboratory Quality Control Center, Xiamen, China; ^3^Institute of Infectious Disease, School of Medicine, Xiamen University, Xiamen, China

**Keywords:** SARS-CoV-2, RT-PCR, false positive, vaccine, contamination

## Abstract

Real-time reverse transcription-polymerase chain reaction (RT-PCR) is the gold standard for diagnosing coronavirus disease 2019 (COVID-19). However, RT-PCR may yield false-positive results, leading to unnecessary countermeasures. Here, we report a “positive” nucleic acid test on a 10-pooled sample during the routine screening that caused many adverse societal effects, and financial and resource losses. However, they were subsequently determined to be a case of vaccine contamination. This case study increases awareness of false-positive RT-PCR results for SARS-CoV-2, especially when participants are vaccinators. Moreover, it could provide relevant suggestions to prevent the recurrence of such incidents.

## Introduction

The coronavirus disease 2019 (COVID-19), caused by severe acute respiratory syndrome coronavirus 2 (SARS-CoV-2), has escalated to a pandemic with devastating morbidity and mortality rates (1). According to the World Health Organization (WHO), the nucleic acid test for SARS-CoV-2, such as real-time reverse transcription-polymerase chain reaction (RT-PCR), is preferred and applicable worldwide for confirming COVID-19 (2). Despite their high sensitivity and specificity, RT-PCR assays are prone to false-negative. A systematic review analysis of the diagnostic sensitivity of SARS-COV-2 RT-PCR found that up to 33% of patients with COVID-19 may have initial false-negative results (3). Much has been written about the issue of false-negative RT-PCR due to differences in sampling site or sample condition, improper sample storage, delayed time to analysis, or low sensitivity of reagents (4–6). Less study has been published regarding the problem of false-positive RT-PCR test. Based on previous experience in investigating false-positive RT-PCR results and discussion with an expert in molecular detection, the two most common problems of contamination and non-specific amplification (e.g., other coronaviruses) can occur during routing COVID-19 screening (7). The false positives have multiple potential adverse effects, such as panic among patients and doctors, and unnecessary waste of resources (8). In China with wide-scale COVID-19 vaccination, false-positive SARS-CoV-2 RT-PCR results have been reported during the environmental surveillance process, which caused a lot of panics (9, 10). The main reasons for this false positive are aerosols generated by medical wastes during or after vaccination that can lead to environmental contamination (9). So far, except for one case study from abroad on throat saliva specimens from suspected patients contaminated by an inactivated SARS-CoV-2 vaccine strain (8), there have been fewer reports of false-positive nucleic acid tests on other human specimens by inhaling vaccine-induced aerosols. Therefore, strategies should be developed to resolve issues if staff members at vaccination premises test false-positive. This is imperative because a false-positive result for nucleic acid tests in a vaccinator's sample may attract increased attention and panic. Here, we report contamination of oropharyngeal swabs obtained from vaccinators with SARS-CoV-2 vaccines. The study may increase awareness of false-positive results for SARS-CoV-2 by RT-PCR, especially when participants are vaccinators.

## Methods

Samples were collected as previously described (11). The 400 μL individual samples were subjected to ribonucleic acid (RNA) extraction using Nucleic Acid Extraction Kit (Magnetic Bead Method) (Tellgen Corporation, China), according to the manufacturer's instructions. The extracted RNA was then subjected to RT-PCR using Da An Detection Kit for 2019-nCoV (Da An Gene Co., Ltd., Sun Yat-sen University, China) that determines the presence of SARS-CoV-2 through identification of *OFR1ab* and *N* genes. When the test yielded positive results, a second highly sensitive ZJ Detection Kit (Shanghai ZJ Bio-Tech Co., Ltd, China), targeting the *OFR1ab, N*, and *E* genes, was used for verification. The RT-PCR assays were performed using the SLAN®-96S Real-Time PCR System (Shanghai Hongshi Medical Technology Co., Ltd., China). RT-PCR for the Vero alpha-satellite sequence was also performed on the extracted DNA samples to detect residual Vero cells in the vaccine. The primers and probes used for amplification are listed in Supplementary Table 1. The cycling conditions were as follows: 55°C for 5 min, 95°C for 30 s, and 45 cycles of 95°C for 5 s, 62°C for 20 s (data collection), and 37°C for 30 s. RT-PCR for Vero cells was performed with the assistance of Xiamen TopBiotech Co., Ltd. (Xiamen, China). Whole-genome sequencing of the original positive 10-pool sample, a positive environmental sample, and the vaccine batch in use were performed as described previously (12). This study was approved by the Institutional Ethics Committee of Zhongshan Hospital, Medical College of Xiamen University, and ethical approval No. is xmzsyyky202196. All studies complied with national legislation and the Declaration of Helsinki guidelines. Informed consent was obtained according to the institutional guidelines.

## Results

### RT-PCR for SARS-CoV-2 in a 10-Pooled Oropharyngeal Swab Sample From Vaccinators

**Figure 1:**
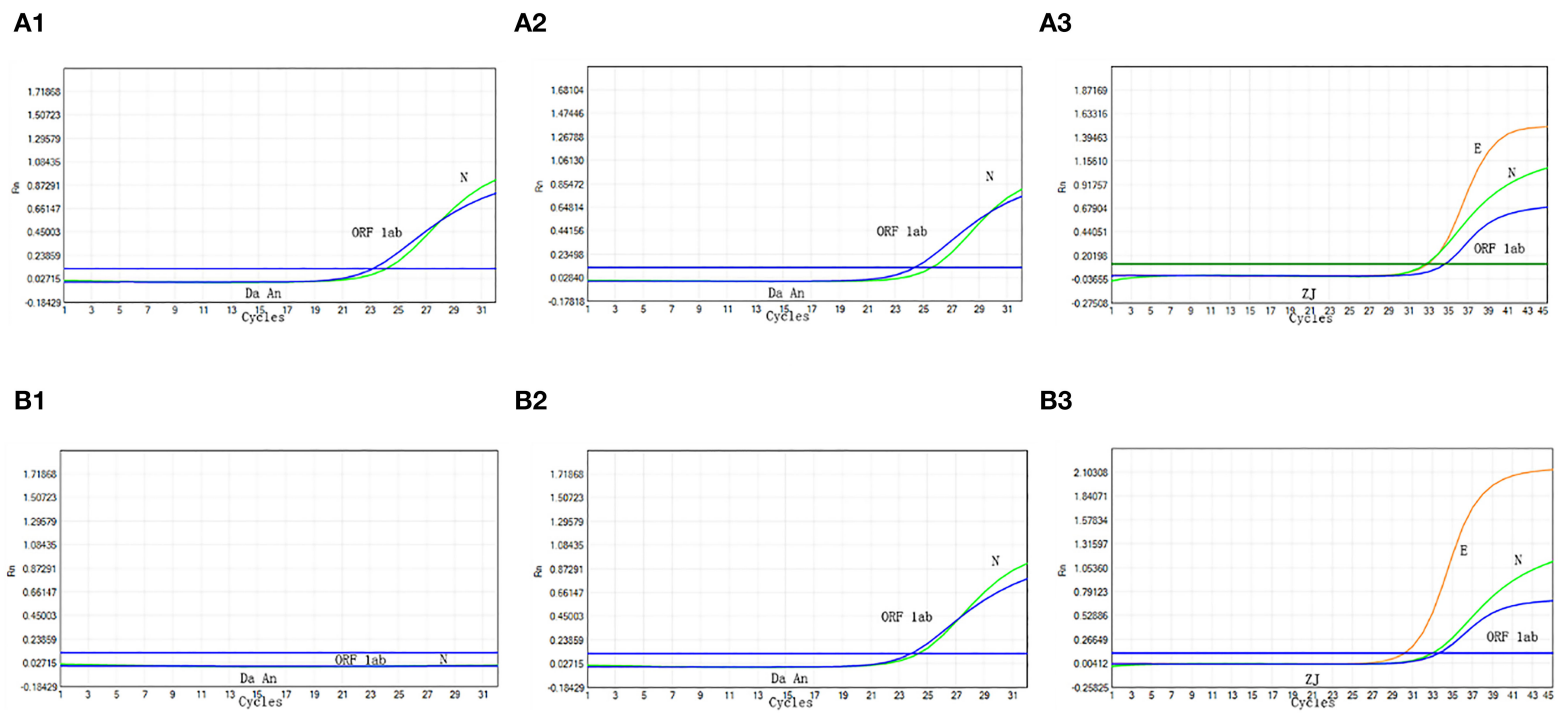
Real-time reverse transcriptase polymerase chain reaction (RT-PCR) analysis of the original 10-pooled oropharyngeal swab sample. The sample is analyzed by RT-PCR with Da An Kit for the first time **(A1)**. The sample is re-extracted and analyzed by Da An Kit **(A2)** and ZJ Kit **(A3)**. The negative process control **(B1)** and positive control **(B2,B3)** are also analyzed with RT-PCR.

During a routine, periodic screening for staff at the Public Health Department on August 1, 2021, in Xiamen, China, we identified a 10-pooled sample that tested positive by Da An Detection Kit [Ct: 23.2 (*N* gene); Ct: 24.1 (*ORF1ab*)] (Figure 1A1). The curves were typical, indicating the possibility of SARS-CoV-2 infection in the samples. Thus, the original specimen was re-extracted and concurrently analyzed using both Da An Kit and ZJ Kit. This specimen also tested positive with both Da An Kit [Ct: 24.3 (*N* gene); Ct: 25.6 (*ORF1ab*)] (Figure 1A2) and ZJ Kit [Ct: 32.5 (*N* gene); Ct: 34.2 (*ORF1ab*); Ct: 33.5 (*E* gene)] (Figure 1A3), thus, validating result of the original test. The negative control and positive control were also analyzed by RT-PCR (Figures 1B1–B3). Therefore, to avoid the further spread of COVID-19, the local CDC immediately informed the 10 vaccinators to maintain home isolation until further tests.

### Single Swab Sample Testing for SARS-CoV-2

Next, different sampling teams immediately approached the homes of the 10 vaccinators and collected single nasopharyngeal swabs twice within 48 h. We then tested the 10 single specimens using the same extraction and RT-PCR detection platform with two different PCR reagents. However, all samples tested negative for SARS-CoV-2 using both reagents, twice. At the same time, these 10 people had no clinical symptoms or potential exposures associated with COVID-19 (see Supplementary Table 2), and until March 20, 2022, ~45 repeated RT-PCR tests (for routine and periodic screening) yielded negative results.

### Nucleic Acid Test for Environmental Surveillance of SARS-CoV-2 at Vaccination Premises

As the 10 individuals were vaccination staff at the same institution, we collected samples from their working environment. Samples from nine places were collected, including six workstation tops and three doorknobs (Table 1). The results showed that the six workstation samples were positive for SARS-CoV-2. The Ct values of *ORF1ab* and *N* genes were close to the original 10-pooled sample using DA Detection Kit, ranging between 17.3 and 24.9 for the *ORF1ab* and between 17.2 and 24.4 for *N* genes. The Ct values of *ORF1ab* and *N* genes detected using ZJ Detection Kit were lower than those of the original 10-pooled samples from the vaccinators. All three doorknob samples tested negative.

**Table 1:** Nucleic acid test for environmental surveillance of Severe Acute Respiratory Syndrome- Coronavirus- 2 (SARS-CoV-2) at vaccination sites.

	**Da An Kit (Ct)**			**ZJ Kit (Ct)**			
**Sampled objects**	**N Gene**	**ORF1ab Gene**	**Interpretation**	**N Gene**	**ORF1ab Gene**	**E Gene**	**Interpretation**
Workstation 1	22.1	23.0	+	30.5	30.9	28.4	+
Workstation 2	20.4	20.7	+	28.6	29.1	26.3	+
Workstation 3	24.4	24.9	+	33.2	33.3	30.4	+
Workstation 4	17.2	17.3	+	25.1	29.1	22.9	+
Workstation 5	22.1	22.3	+	30.1	30.2	27.8	+
Workstation 6	20.4	21.1	+	29.0	29.4	26.7	+
Doorknob 1	-	-	-	-	-	-	-
Doorknob 2	-	-	-	-	-	-	-
Doorknob 3	-	-	-	-	-	-	-

### Vero Alpha-Satellite Sequence RT-PCR and Genome Sequence Comparison of Virus Genome Sequence in the Positive Samples and Inactivated Vaccine Strain of SARS-CoV-2

As the SARS-CoV-2 isolates in inactivated vaccines were passaged in Vero cells, we suspected that the Vero cell sequence may also be found in the false-positive DNA samples and inactivated SARS-CoV-2 vaccine. Therefore, we performed Vero alpha-satellite sequence RT-PCR. The analyses suggested that the original 10-pooled sample, environmental samples from Workstation 4, and vaccine used tested positive for Vero alpha-satellite sequence, whereas nucleic acid samples from real-positive SARS-CoV-2 RT-PCR samples and control human samples tested negative for this sequence. Furthermore, whole viral genome Nanopore sequencing was performed for the original 10-pooled sample, environmental samples from Workstation 4, and the same batch of vaccines used previously. The data indicated that the viral genome sequences in the original 10-pooled sample and environmental samples were consistent with the vaccine strains, and there were no significant differences in the genomic variation site of samples from the local 10-pooled case found on August 01, 2021 (data not shown).

## Discussion

As of November 28, 2021, over 279 million people have been infected with SARS-CoV-2 worldwide, representing an unimaginable rapid spread of the disease. In China, the “Four Early” technical guideline is the primary countermeasure in the prevention and control of COVID-19, of which “early detection” plays an irreplaceable role and is achieved with periodic nucleic acid screening. When a positive case occurs, especially in a low-prevalence setting, emergency measures are taken immediately (13). Therefore, the accuracy of diagnosis is imperative, as it can reduce the spread of disease and avoid financial and resource losses, unnecessary public isolation, adverse psychological pressures, and societal effects.

In this study, we identified a “false-positive” case of COVID-19 due to contamination of specimens by an inactivated vaccine from vaccinators. There have been many cases of false-positive results in the examination of pathogens due to vaccines, especially inactive vaccines (14, 15). Waibel et al. found detectable viruses on dressings covering smallpox vaccination sites (14). Fan et al. found that hemostatic stickers used after SARS-CoV-2 vaccine inoculation have a risk of nucleic acid contamination (10). The present study is another example of a rare false-positive RT-PCR test for SARS-COV-2. The significance of these false-positive results is far-reaching, particularly with effects on procedures for handling positive nucleic acid test results from vaccinators.

The COVID-19 vaccine has been vaccinated throughout China. As of March 20, 2022, 3.226 million doses of the COVID-19 vaccine have been administered. Therefore, a large number of staff members of the Public Health Department are working on the frontline to assist vaccination departments and undergo regular nucleic acid screening. These staff members contact several individuals every day, and thus, are at high risk of exposure and transmission of infection. The “false positive event” detected in such vaccinators is not a mere interpretation of the experiment, but they correspond to more severe emergency countermeasures (16). As early as January 2021, a testing organization found that nucleic acid tests for the environmental surveillance of SARS-CoV-2 in vaccination sites showed positive results. Once reported, this caused panic in society, and people mistakenly believed that the vaccination staff might be infected with COVID-19. Finally, it was confirmed the positive environmental samples were caused by vaccine contamination (14). Subsequently, the same problem also happened in environmental samples from other vaccination sites. Therefore, the Chinese CDC issued a document requiring that regular nucleic acid tests for the environmental surveillance of SARS-CoV-2 are not recommended to avoid unnecessary social panic and depletion of resources. Furthermore, employees and vaccine injectors are required not to perform SARS-CoV-2 nucleic acid testing within 48 hr after vaccination, to avoid false-positive caused by vaccine contamination (17).

It may be difficult to recognize that a positive RT-PCR result is false-positive. First, suspected cases can be preliminarily judged based on epidemiological history and clinical manifestations. In the absence of either of these characteristics, there is a possibility of false-positive results, especially for vaccinators or vaccine subjects within 48 h. In such situations, the original sample should be re-extracted and re-tested. If still positive, a new sample should be obtained and tested (18). If contamination with the inactivated vaccine is suspected, the residual Vero cell sequence is recommended for testing. When conditions permit, genome sequencing is recommended to determine whether the nucleic acid sequence is from a vaccine strain (8). Moreover, with a simultaneous increase in the number of cases in local areas, it is necessary to conduct comprehensive research based on epidemiological and viral genome sequencing data. If the test is positive for SARS-CoV-2 due to vaccine contamination, relevant departments should avoid excessive countermeasures to reduce unnecessary economic losses.

The limitations of the present study should be acknowledged. First, the sample size is relatively small. In the future, it can be considered to add samples from different vaccination centers around different cities or countries to see whether the result is the same. Secondly, there is a lack of analysis on the time (half-life) of workers and vaccine injectors carrying COVID-19 vaccine aerosol. After knowing the half-life, there is a good reference for the sampling time of nucleic acid detection in this kind of population. Another limitation was that we did not analyze the vaccinators' work time as an influencing factor in this study. The duration of the vaccinators' shifts may alter the rate of a false-positive result.

In conclusion, the detection of viral RNA in nasopharyngeal/oropharyngeal swabs is the gold standard for the diagnosis of COVID-19. However, contamination by the vaccine may affect RT-PCR results, leading to false-positive results in individual swab samples and causing unnecessary panic. Therefore, the presence of the following factors should be eliminated with appropriate measures: (1) the environmental management of vaccination sites should be strengthened, especially ventilation, and the waste materials should be segregated and treated separately; (2) strict measures should be adopted to protect staff by minimizing the inhalation of vaccine aerosols; (3) before sampling, the vaccinators should stop working for 24 h, and clean the nasopharynx and throat; (4) to remove aerosols at vaccination sites over time, the environment should be disinfected with both chlorine-containing disinfectants and commercial nucleic acid removers; and (5) if possible, professional training of vaccinators and relevant guidelines should be implemented to ensure highly effective and precise countermeasures.

## Data Availability Statement

The raw data supporting the conclusions of this article will be made available by the authors, without undue reservation.

## Ethics Statement

The studies involving human and animal participants were reviewed and approved by Institutional Ethics Committee of Zhongshan Hospital, Medical College of Xiamen University. Written informed consent to participate in this study was provided by the participants or their legal guardian/next of kin.

## Author Contributions

X-QK and M-LT wrote the manuscript with the support of T-CY. X-QK, Y-JW, Z-XF, and M-LT performed the PCR detection and genome sequencing. T-CY has provided guidance and advice as a senior scientist and consultant during routine screening of COVID-19 infection. All authors have performed a literature search and reviewed and provided revisions to the final draft of the manuscript.

## Funding

This work was supported by the National Natural Science Foundation of China [Grant Numbers 81772260, 81973104, and 81871729], Key Projects for Province Science and Technology Program of Fujian Province [Grant Number 2018D0014]. The funders played no role in the study design, data collection, analyses, the decision to publish, or manuscript preparation.

## Conflict of Interest

The authors declare that the research was conducted in the absence of any commercial or financial relationships that could be construed as a potential conflict of interest.

## Publisher's Note

All claims expressed in this article are solely those of the authors and do not necessarily represent those of their affiliated organizations, or those of the publisher, the editors and the reviewers. Any product that may be evaluated in this article, or claim that may be made by its manufacturer, is not guaranteed or endorsed by the publisher.
