# Supplementary material for: False-Positive Results of SARS-CoV-2 RT-PCR in Oropharyngeal Swabs From Vaccinators
Source: Front Med (Lausanne). 2022 Jun 10;9:847407. doi: 10.3389/fmed.2022.847407 (PMC9226675; doi:10.3389/fmed.2022.847407)
Supplement: Supplementary file 1 [file Table_1.pdf]

Table S1. The primers and the probe sequences used in the study.

Primer	Orginal Name Sequence 5′–3′
β-actin F primer	5′-CTCTGTGTTCTGTTAATTCATCTCAC-3′
β-actin R primer	5′-CCAAATATCCCTTTGCCAATTCC-3′
Probe	5′-AGAACAGCCTTAGCGAAAGGCTT-3′
