# Supplementary material for: False-Positive Results of SARS-CoV-2 RT-PCR in Oropharyngeal Swabs From Vaccinators
Source: Front Med (Lausanne). 2022 Jun 10;9:847407. doi: 10.3389/fmed.2022.847407 (PMC9226675; doi:10.3389/fmed.2022.847407)
Supplement: Supplementary file 2 [file Table_2.pdf]

Table S2. Demographic information of ten vaccinators in the study.

No.	Gender	Age	Symptoms[&]	Potential exposures[&]
1	Female	25	No	No
2	Female	31	No	No
3	Female	28	No	No
4	Female	28	No	No
5	Male	26	No	No
6	Female	33	No	No
7	Female	25	No	No
8	Female	27	No	No
9	Female	45	No	No
10	Female	39	No	No

[&] Clinical symptoms or potential exposures associated with COVID-19.
